# Supplementary material for: High-pressure crystallography shows noble gas intervention into protein-lipid interaction and suggests a model for anaesthetic action
Source: Commun Biol. 2022 Apr 14;5:360. doi: 10.1038/s42003-022-03233-y (PMC9010423; doi:10.1038/s42003-022-03233-y)
Supplement: Supplementary file 2 — Supplementary Materials [file 42003_2022_3233_MOESM2_ESM.pdf]

Supplementary Materials

Figures

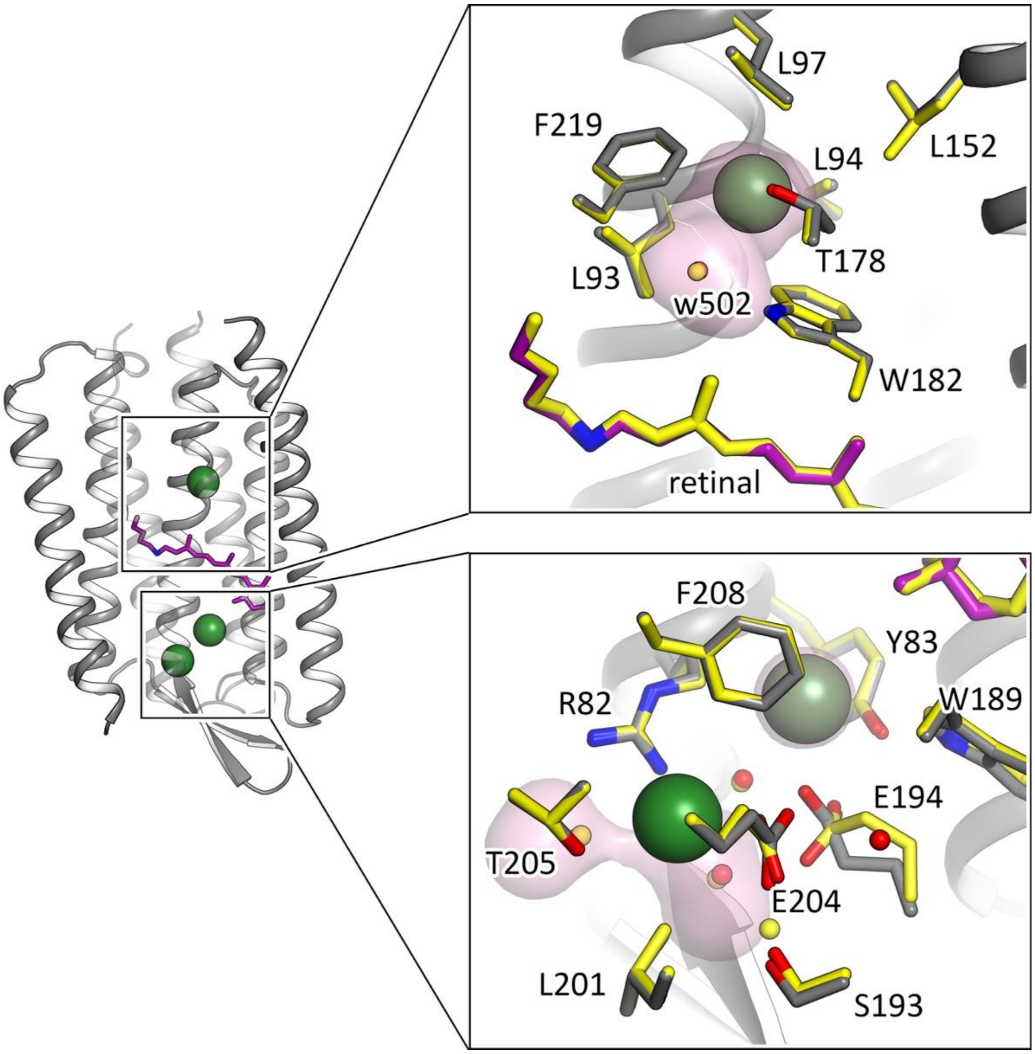

a, tmBR-argon

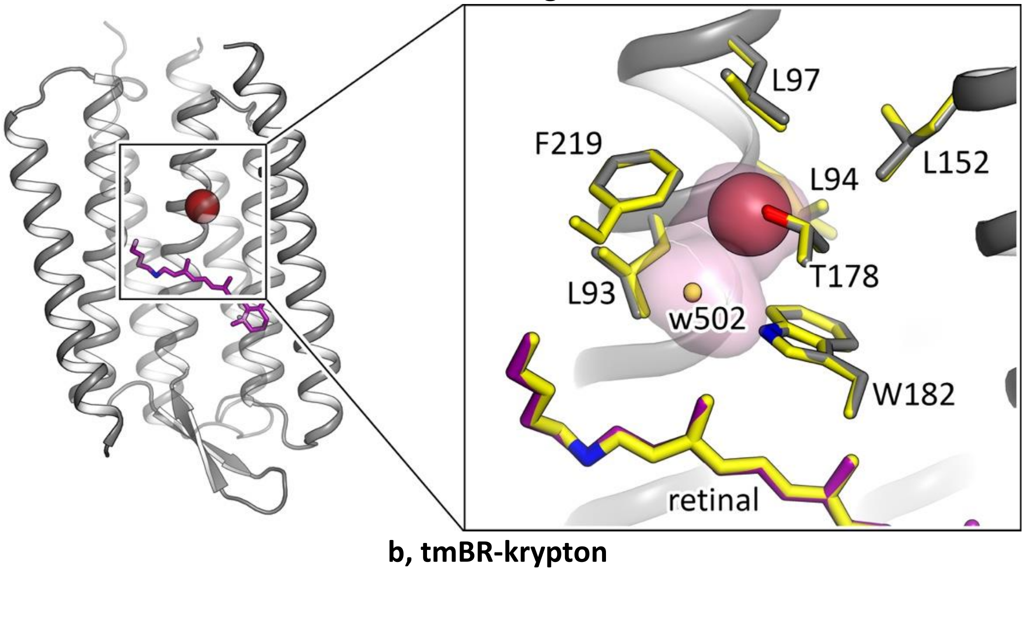

b, tmBR-krypton

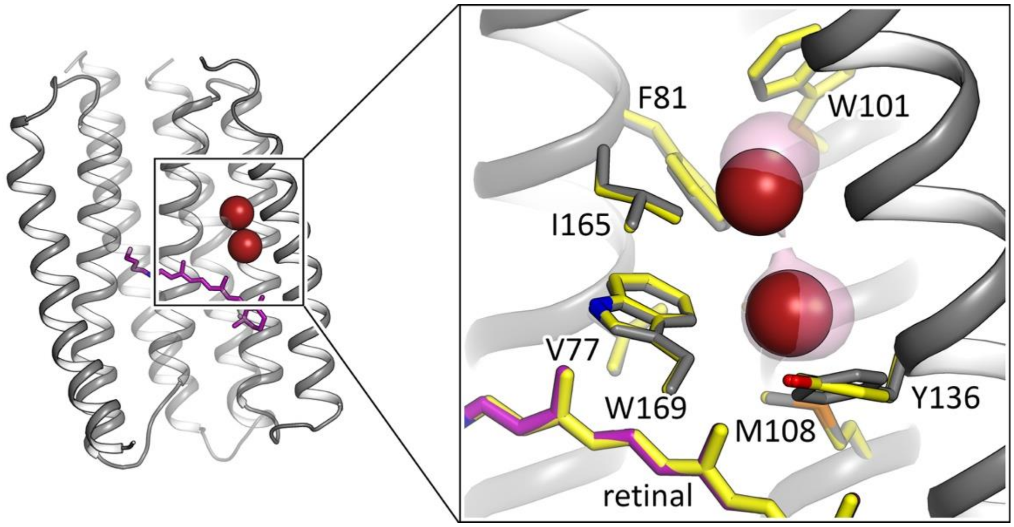

c, MAR

Supplementary Figure 1. Schematic illustration of the positions of the internal noble gas atoms and their environments in the structures of tmBR (a, argon; b, krypton) and MAR (c, krypton). The protein structures are shown in grey cartoon representation, retinal molecules are coloured in purple, krypton atoms are coloured in red whereas argon atoms are coloured in green. Panels on the right illustrate noble gas atom environment and compare side chain arrangement with a native structure in those regions (shown in yellow). Red transparent surface shows internal voids (calculated by HOLLOW[72] with default parameters, see Methods) in protein structure.

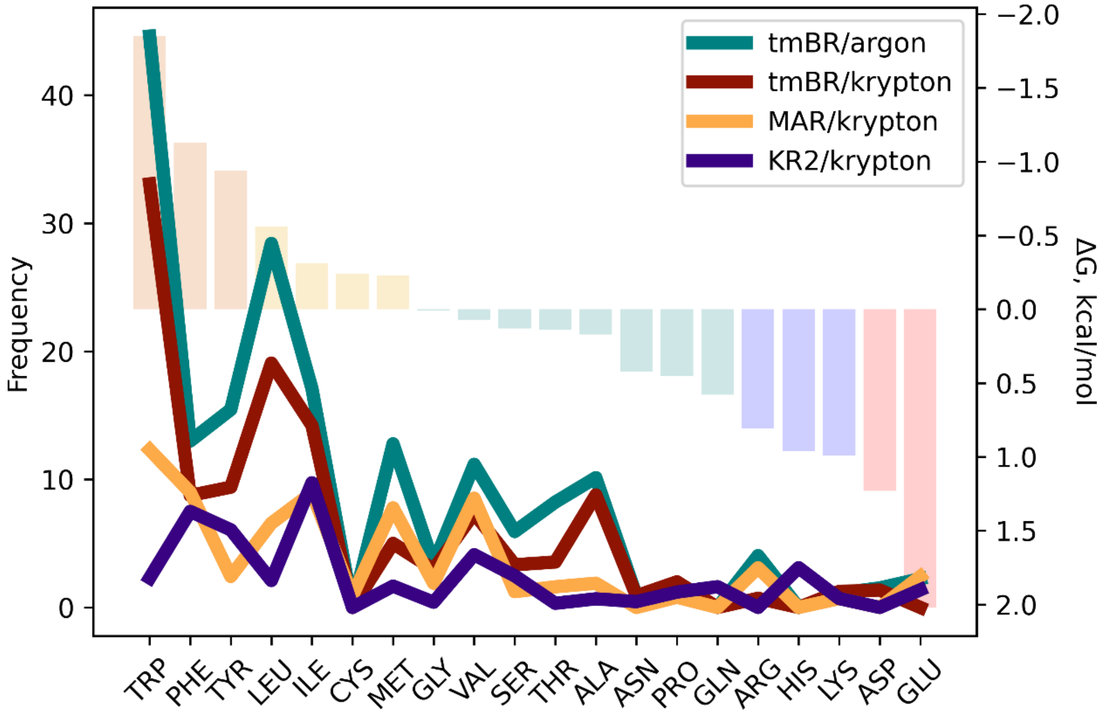

Supplementary Figure 2. Frequency of each residue in a noble gas atom environment. The value of frequency was calculated as the number of atoms of each particular residue in a 5Å-vicinity of noble gas atoms, normalised to the frequency of natural occurrence of this residue[90]. An empirical hydrophobicity scale is shown at the background and depicts free energy ΔG required to put a residue into the core of lipid bilayer[91] (right scale).

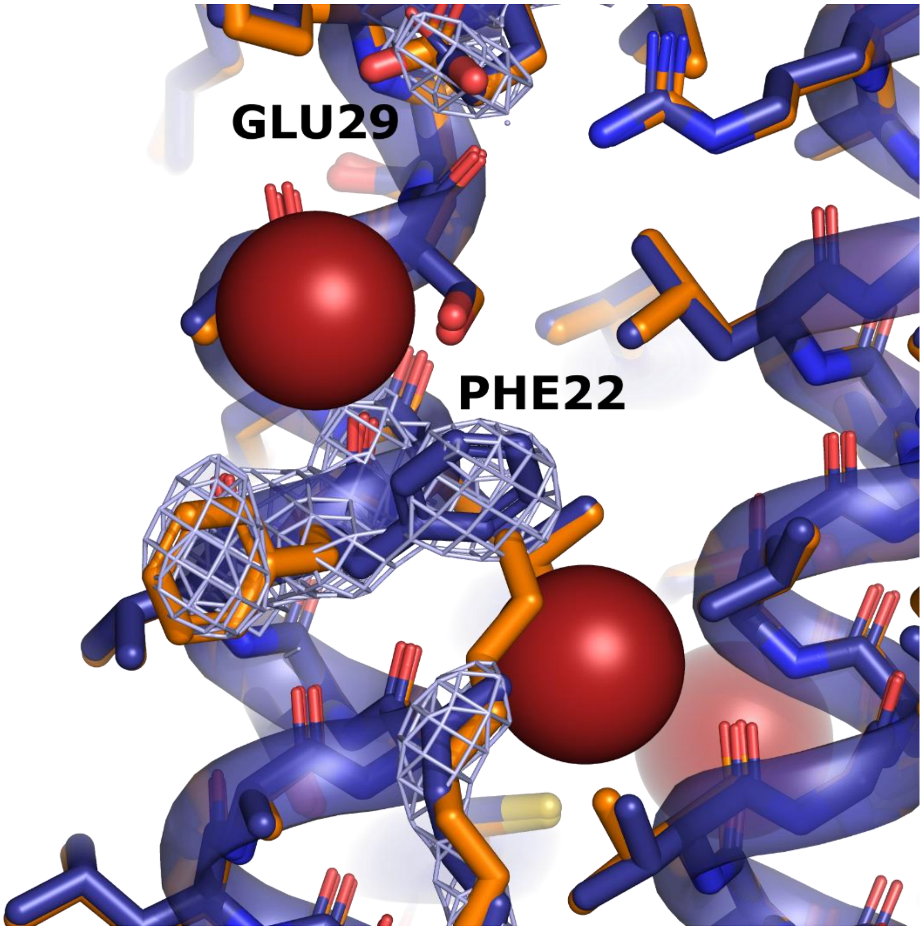

Supplementary Figure 3. Structural changes in MAR upon binding of krypton atoms. The structure of MAR-Kr is coloured in blue, whereas the corresponding native structure (aligned to MAR-Kr structure by C_α positions) is coloured in orange. The electron density map ($2F_o$-F_c) of the derivative data set is drawn in light blue at 1.2 r.m.s. level around residues of interest and clearly shows their alternative conformation (PHE22, GLU29, and a lipid fragment) relative to the native structure. Krypton atoms are shown as red spheres.

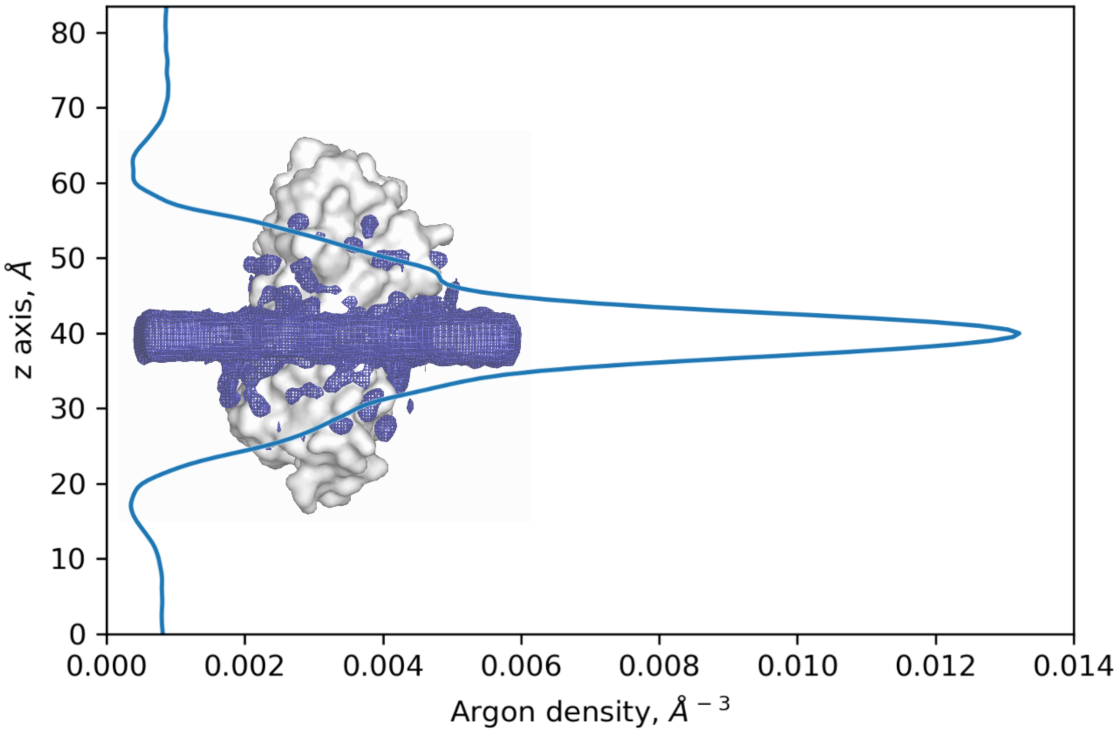

Supplementary Figure 4. Simulated argon density at the level of 3x{map mean value} on the surface of tmBR and corresponding mean density distribution along z axis (perpendicular to the bilayer) show that noble gas atoms segregate in the core of lipid bilayer.

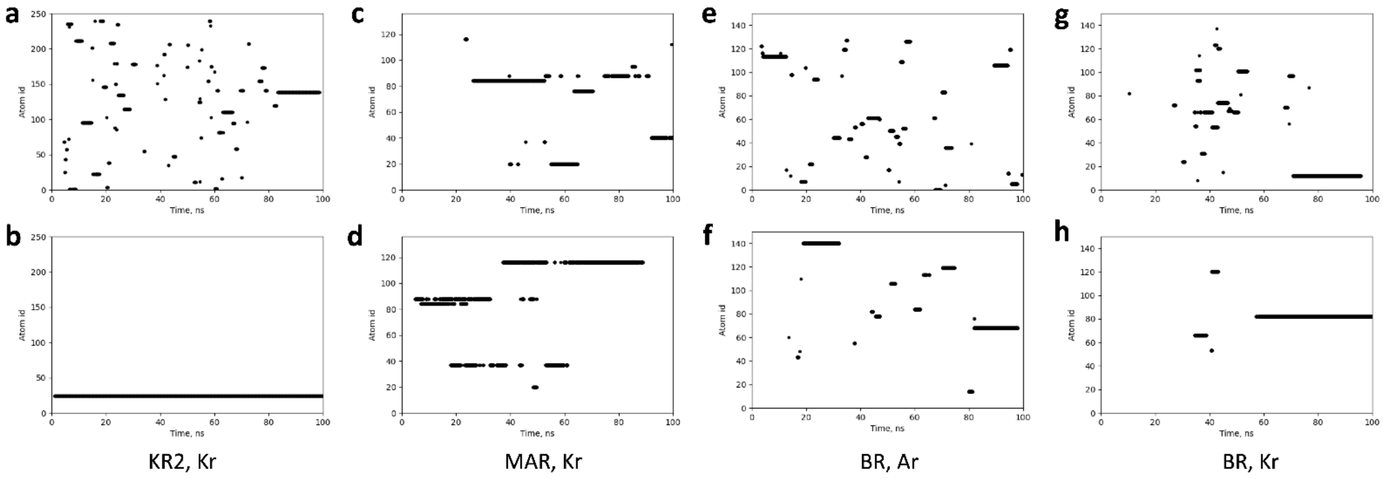

Supplementary Figure 5. Analysis of binding events at representative sites in the simulated systems. Black dots indicate that the corresponding gas atom is bound at the site. (a-b) KR2-krypton; (c-d) MAR-krypton; (e-f) tmBR(BR)-argon; (g-h) tmBR(BR)-krypton.

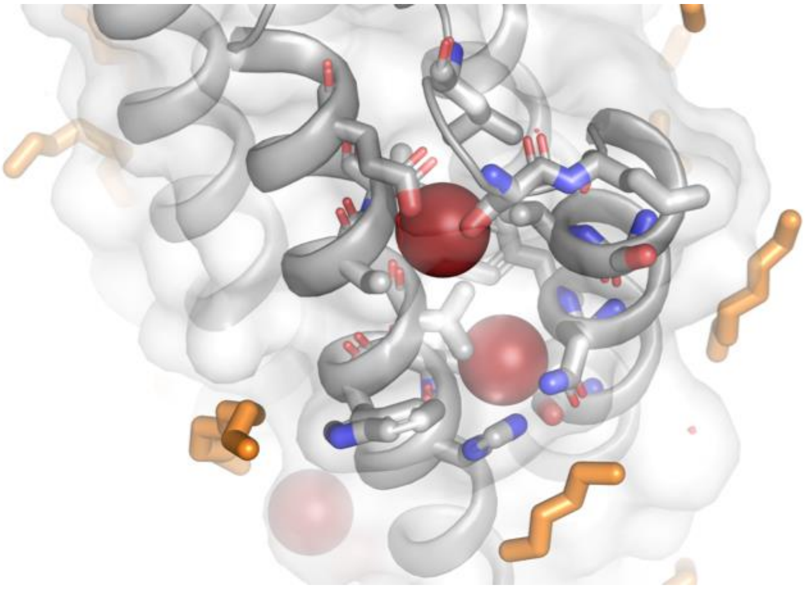

Supplementary Figure 6. Krypton atoms (red spheres) trapped in the cleft between α-helices of KR2. Nearby amino acid side chains (shown as grey sticks) are blocking access to these clefts. Lipid fragments are shown as orange sticks.

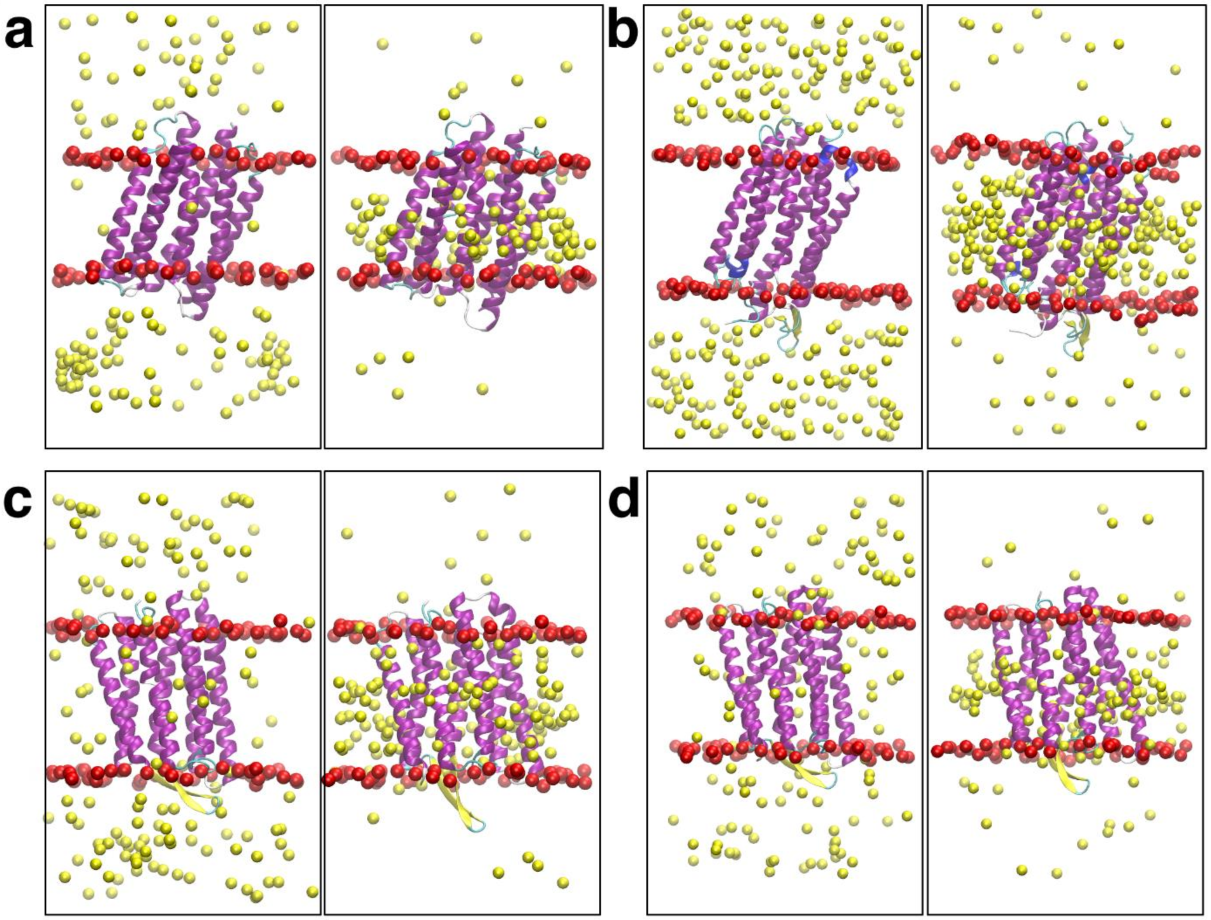

Supplementary Figure 7. Representative snapshots of the initial (each panel, left) and final (each panel, right) configurations of the simulated systems: MAR (a), KR2 (b), tmBR with krypton (c), and tmBR with argon(d). The protein molecules are shown using cartoon representation coloured accordingly to the secondary structure; noble gas atoms are shown as yellow spheres; phosphorus atoms of the membrane are shown as red spheres. Water, ions, and all lipid atoms except phosphorus are omitted for clarity.

Supplementary Text

Internal binding sites and their relevance to rhodopsin function

Noble gases helped us to visualize small hydrophobic cavities often found in the cytoplasmic inner parts of microbial rhodopsins. The role of such cavities, however, remains poorly understood[92]. One of the hypotheses is that these cavities can accommodate diffuse water molecules, which are relocated during the photocycle and thus may play the key role in functioning of a rhodopsin. As a demonstration of this concept, in the early L intermediate state of BR the continuous chain of five water molecules was predicted to be formed between the retinal Schiff base and the proton donor, D96[93]. However, in the ground state only two water molecules were identified in the cytoplasmic inner part of the protein[94]. At the same time, we observe in our work a small cavity near T178 residue and HOH502 molecule occupied by a krypton atom in the tmBR-krypton derivative structure. This cavity is suitable for accommodation of an additional water molecule taking into consideration the distances to nearby residues and the cavity volume.

Another possibility is that this cavity near helix E may be a part of ion pathway. Indeed, in the light-driven chloride pump halorhodopsin from *Natronomonas Pharaonis*, the channel containing a chain of water molecules is transiently formed in this region in the N-intermediate state[95]. This channel was suggested to be a chloride-release pathway[95].

Inline with the described observations, the absence of krypton atoms inside the light-driven sodium pump KR2 protomer supports both the absence of hydrophobic cavities inside the protein and the lack of their need for sodium pumping. Indeed, while the extracellular inner part is polar as in many other microbial rhodopsins, the cytoplasmic part also contains a large polar ion-uptake cavity filled with water molecules already in the ground state[46,58,96]. The recently reported structure of the O-state of KR2[96,97] revealed that the cytoplasmic region is completely restored in the intermediate. This latter observation together with the absence of the pronounced M-state, which presumably corresponds to the sodium translocation through the cytoplasmic side of the protein, also suggest the absence of large conformational changes in

the cytoplasmic inner part of KR2 during photocycle. All this makes the presence of additional hydrophobic cavities or/and water molecules in the region unnecessary.

Finally, yet importantly, in the tmBR-argon structure we observed two argon atoms in the extracellular part of the protein. These atoms interact with the R82 residue, which is a key determinant of the vectorial proton translocation[98], and are located close to the E194 and E204, which constitute the so-called ‘proton release group’ (PRG) of BR[98]. The binding of argon also causes notable rearrangements of the PRG. This observation supports the high flexibility of this region, which was hypothesized and demonstrated recently by the identification of two conformations in the ground state structure of BR[99]. Thus, presence of argon may stabilize one of these conformations, presumably the minor one, which was not identified in the native structure because of lack of resolution.

References

90. King, J. L. & Jukes, T. H. Non-Darwinian Evolution: Most evolutionary change in proteins may be due to neutral mutations and genetic drift. Science 164, 788–798 (1969).

91. Wimley, W. C. & White, S. H. Experimentally determined hydrophobicity scale for proteins at membrane interfaces. Nat. Struct. Mol. Biol. 3, 842–848 (1996).

92. Matthews, B. W. & Liu, L. A review about nothing: Are apolar cavities in proteins really empty? Protein Sci. NA-NA (2009) doi:10.1002/pro.61.

93. Maeda, A., Gennis, R. B., Balashov, S. P. & Ebrey, T. G. Relocation of Water Molecules between the Schiff Base and the Thr46−Asp96 Region during Light-Driven Unidirectional Proton Transport by Bacteriorhodopsin: An FTIR Study of the N Intermediate †. Biochemistry 44, 5960–5968 (2005).

94. Luecke, H., Schobert, B., Richter, H.-T., Cartailler, J.-P. & Lanyi, J. K. Structure of bacteriorhodopsin at 1.55 Å resolution. J. Mol. Biol. 291, 899–911 (1999).

95. Kouyama, T., Kawaguchi, H., Nakanishi, T., Kubo, H. & Murakami, M. Crystal Structures of the L 1 , L 2 , N, and O States of pharaonis Halorhodopsin. Biophys. J. 108, 2680–2690 (2015).

96. Kovalev, K. et al. Molecular mechanism of light-driven sodium pumping. Nat. Commun. 11, 2137 (2020).

97. Skopintsev, P. et al. Femtosecond-to-millisecond structural changes in a light-driven sodium pump. Nature 583, 314–318 (2020).

98. Gerwert, K., Freier, E. & Wolf, S. The role of protein-bound water molecules in microbial rhodopsins. Biochim. Biophys. Acta BBA - Bioenerg. 1837, 606–613 (2014).

99. Hasegawa, N., Jonotsuka, H., Miki, K. & Takeda, K. X-ray structure analysis of bacteriorhodopsin at 1.3 Å resolution. Sci. Rep. 8, 13123 (2018).
