# Supplementary material for: High-pressure crystallography shows noble gas intervention into protein-lipid interaction and suggests a model for anaesthetic action
Source: Commun Biol. 2022 Apr 14;5:360. doi: 10.1038/s42003-022-03233-y (PMC9010423; doi:10.1038/s42003-022-03233-y)
Supplement: Supplementary file 3 — Description of Additional Supplementary Files [file 42003_2022_3233_MOESM3_ESM.pdf]

Description of Additional Supplementary Files

File name: Supplementary Video 1
Description: Arrangement of peaks of the anomalous difference map of argon (in green, sampled at 3.5 r.m.s. level) and the corresponding MD map (in blue, sampled at the level of {mean + 3.5×std.dev.}) on the surface of tmBR molecule. Neighbouring tmBR molecules in crystal lattice are shown in transparent dark.

File name: Supplementary Video 2
Description: Arrangement of peaks of the anomalous difference map of krypton (in red, sampled at 3.5 r.m.s. level) and the corresponding MD map (in blue, sampled at the level of {mean + 3.5×std.dev.}) on the surface of tmBR molecule. Neighbouring tmBR molecules in crystal lattice are shown in transparent dark.

File name: Supplementary Video 3
Description: Arrangement of peaks of the anomalous difference map of krypton (in red, sampled at 3.5 r.m.s. level) and the corresponding MD map (in blue, sampled at the level of {mean + 3.5×std.dev.}) on the surface of KR2 molecule. Neighbouring KR2 molecules in crystal lattice are shown in transparent dark.

File name: Supplementary Video 4
Description: Arrangement of peaks of the anomalous difference map of krypton (in red, sampled at 3.5 r.m.s. level) and the corresponding MD map (in blue, sampled at the level of {mean + 3.5×std.dev.}) on the surface of MAR molecule. Neighbouring MAR molecules in crystal lattice are shown in transparent dark.
